# Larvicidal Potential of Five Selected Dragonfly Nymphs in Sri Lanka over* Aedes aegypti* (Linnaeus) Larvae under Laboratory Settings

**DOI:** 10.1155/2018/8759459

**Published:** 2018-12-03

**Authors:** Chathurika Samanmali, Lahiru Udayanga, Tharaka Ranathunge, Sandun J. Perera, Menaka Hapugoda, Chathura Weliwitiya

**Affiliations:** ^1^Department of Natural Resources, Faculty of Applied Sciences, Sabaragamuwa University of Sri Lanka, Sri Lanka; ^2^Department of Biosystems Engineering, Faculty of Agriculture & Plantation Management, Wayamba University of Sri Lanka, Sri Lanka; ^3^Department of Biosystems, Faculty of Agriculture & Plantation Management, Wayamba University of Sri Lanka, Sri Lanka; ^4^HELPO Eco Green Ltd., Talbot Town, Galle, Sri Lanka

## Abstract

**Introduction:**

Limitations in breeding source reduction practices, development of insecticide resistance in mosquitoes, and ill effects of chemical controlling methods on human and ecosystem health have motivated Sri Lankan authorities working for dengue control to seek for alternative, ecofriendly, and sustainable approaches for controlling of* Aedes* vectors, to manage dengue epidemics. The present study attempted to investigate the predation efficiency of locally available dragonfly nymphs over* Aedes aegypti *under laboratory conditions, aiming to evaluate the potential of using dragonflies as biocontrol agents against dengue.

**Methods:**

Nymphal stages of five locally abundant dragonfly species were collected from different stagnated water bodies in Belihuloya area. After morphological identification, a well grown individual of each species was starved for 12 hours and introduced into a glass tank containing 1L of pond water with 200 larvae (4th instar) of* Aedes aegypti*. Number of larvae survived in the tank was enumerated hourly up to 48 hours. In case where >75% of larvae are consumed by dragonfly nymphs, additional* Ae. aegypti* larvae were introduced into such tanks. Experiment was repeated for five times. Same procedure was followed with different stages of growth of the dragonfly nymphs characterized by the highest predation rate. General Linear Model followed by Tukey's pairwise comparison was used for statistical analysis.

**Results:**

The predation rates of different dragonfly species varied significantly (p<0.05), whereby* Anax indicus* (110±7.14 per day) indicated the highest, followed by* Pantala flavescens *(54.07±5.15) and* Gynacantha dravida *(49.00±11.89), while* Tholymis tillarga *(23.47±2.48) had the lowest. Further, significant variations in the larval predation were found among different maturity stages (10–20; 25-35; and 35–45 mm in body length) of* Ana. indicus *(p<0.05). Regardless of statistical significance, a relatively higher larvicidal activity was observed at dusk than in dawn.* Conclusion. Ana. indicus*, which is characterized by the highest predation rate, and* P. flavescens *that has the widest geographical distribution within Sri Lanka along with a notable predation efficacy could be recommended as potential candidates for field trials in biological control of dengue outbreaks via suppression of* Ae. aegypti *larvae.

## 1. Background

Mosquitoes pose one of the main hazards to human health as they perform a major role in the transmission of vector borne diseases [1]. Among them, dengue is a fast growing mosquito-borne viral disease, which is widely spreading over the world. About 50 million dengue viral infections occur every year and virtually 2.5 billion people live in dengue endemic countries [2]. Approximately, 1.8 billion people (more than 70%) are at risk for dengue viral infection within the member states of the World Health Organization's Western Pacific and South-East Asia Regions, which contribute to approximately 75% of the recent global disease burden due to Dengue Fever [3].

Sri Lanka has been affected by epidemics of Dengue Fever (DF) and Dengue Haemorrhagic Fever (DHF) for over two decades. In Sri Lanka,* Aedes aegypti* remain as the primary vector supported by* Ae. albopictus* as the secondary vector. Dengue viral infections have been reported from Sri Lanka since the mid-1960s. DF was serologically corroborated in 1962 and the existence of DF in all of the major towns situated below 1200 m elevation was confirmed within the period of 1976–1978 [4]. With its change in serotype(s), an alarming increase in the incidence of dengue is at play in Sri Lanka, causing the highest ever number of dengue cases as 186, 101 with over 440 deaths in 2017 [5].

Absence of effective drugs and vaccines for the four serotypes of dengue virus has restricted the efficacy of patient management approaches, making vector control and management as the most practical option to control dengue within Sri Lanka. For this, a variety of approaches such as environmental management, chemical control and biological control, etc. are being considered recently. Different, chemical insecticides are being widely used to control adult and larval mosquitoes for many decades, within many countries including Sri Lanka. However, the unintended side effects on human and ecosystem health, development of resistance within mosquitoes, and prominent financial costs have resulted in notable failures in use of chemical based controlling approaches such as larvicides, long-lasting insecticidal nets, Indoor Residual Spraying (IRS), etc. [6–8]. Therefore, authorities working for dengue control seek for alternative, innovative, and ecofriendly methods to reduce both larval and adult vector densities [9]. Among numerous novel strategies, such as Sterile Insect Technique (SIT) and Incompatible Insect Technique (IIT), controlling aquatic larval stages of vector mosquitoes by using their indigenous natural enemies (pathogens, parasites, and predators), also known as biological control, remain as one of the low cost and ecofriendly approach of integrated vector control [9].

Biological control methods attempt to utilize the natural enemies of mosquitoes at different stages of life cycle, both as predators and parasites. A great diversity of living organisms including microbes, fungi, protozoa, nematodes, other invertebrates, and vertebrate predators have been recognized as potential mosquito control agents [10, 11]. Among different biological controlling agents, predatory insects like damselfly and dragonfly nymphs have gained a high consideration as significant predators of many microinvertebrates including the larvae of* Aedes* mosquitoes [11–13]. Both damselflies and dragonflies belong to Order Odonata, specifically into suborders Zygoptera and Anisoptera, respectively. At present, a total of 124 species, consisting of 66 species belonging to the suborder Anisoptera and 58 species into the suborder Zygoptera, have been reported from Sri Lanka [14].

Both, the nymph and the adult of these two suborders predate on mosquitoes [1]. High reproduction capacity, adaptability to the introduced environment, preference for the target pest population in the existence of substitute natural prey, and overall interaction with native organisms have been recognized as key characteristics of any biological controlling agent [9]. Among different food preferences of dragonfly nymphs, small larval forms such as* Aedes* larvae remain preferred by them even in their natural habitats, while adult dragonflies also predate on adult mosquitoes [15, 16]. Further, Odonates bear no harmful impacts on the humans [17, 18]. Many countries in the world, especially in the South-Asian region, have evaluated the practical efficacy of using nymphal Odonates as mosquito control agents. Myanmar [19] and India [12, 20] have successfully used a variety of dragonflies as a potential biological resource in regulating the larval populations of vector and pest mosquitoes [18].

Since, 1960s the authorities working for dengue control in Sri Lanka have focused more on the chemical based vector management, while the effort on biological controlling of* Aedes* is only limited to larvicidal feasibility assessments of few copepod and fish species [21]. Regardless of the remarkable diversity and wide distribution of dragonflies within the country, Sri Lanka has not paid any attention to the dragonflies as a potential biocontrol agent of* Aedes* mosquitoes. With realization of the restrictions in current vector controlling activities, the country should move towards simple, efficient, and ecofriendly methods of vector management. Therefore, the current study was devised to evaluate the potential of using dragonflies as a biocontrol agent to suppress the vector population of* Ae. aegypti* aiming towards management of dengue epidemics within the country.

## 2. Methods

### 2.1. Establishment of* Aedes aegypti* Colony

An adult mosquito collection was conducted in the Narangodapaluwa Medical Officer of Health (MOH) area, Ragama, and captured mosquitoes were transported to the laboratory for mass rearing at the Molecular Medicine Unit, Faculty of Medicine, University of Kelaniya, Sri Lanka. Within the laboratory, only the* Ae. aegypti *mosquitoes were separated through morphological identification by well-trained entomologists. Eggs laid by a single* Ae. aegypti* blood-engorged female were used to establish a mosquito colony of* Ae. aegypti. *Each colony was maintained in 24 x 24 x 24 cm cages with mesh screening on top, under a 12:12 (light:dark) cycle at standard conditions (at 27 ± 2°C and 75 ± 5% humidity), adhering to the standard protocols suggested by Gunathilaka* et al.* [22, 23]. Cattle blood was used to feed the mosquitoes through the metal plate feeding technique [23].

The eggs laid by them were allowed to be hatched within 2-3 days after oviposition. The first instar larvae (L_1_) were transferred daily from the oviposition cups to plastic trays (40 × 25 × 6 cm), with 1,000 ml of water, while maintaining a larval density of 750-800 individuals per tray. Approximately 1 ml of a standard larval diet containing tuna meal (50%), bovine liver powder (36%), and yeast (14%), was added to the larval trays twice a day until the larvae were reared up to the 4th instar stage (L_4_) [23]. Thence formed 4th instar larvae were used for the predation trials as described below.

### 2.2. Establishment of Dragonfly Nymph Colonies

**Figure 1:**
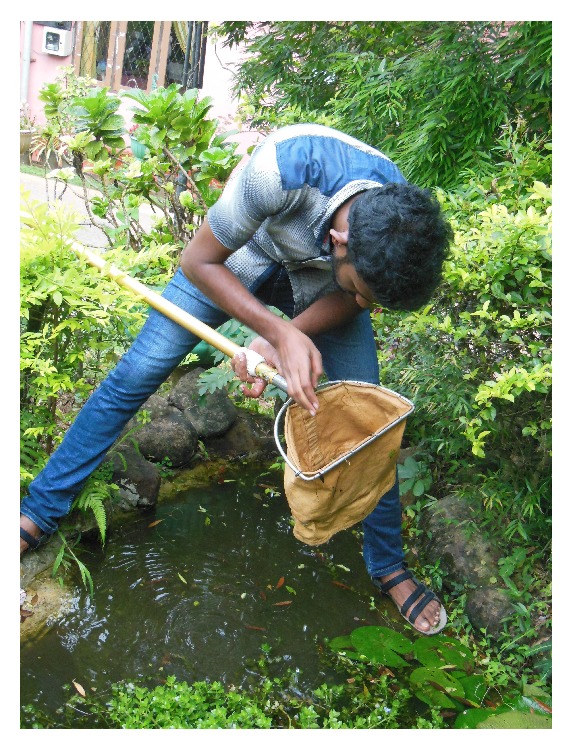
Collection of dragonfly larvae with the D-framed net.

Dragonfly nymphs were collected from different stagnated water bodies located within the premises of the Sabaragamuwa University of Sri Lanka in Belihuloya. They were collected using a D-framed benthic hand dip net at a depth of one to two feet in stagnated water (Figure 1). The collected nymphs were recorded and transferred into sampling jars provided with water from the same waterbody with water weeds and leaf litter. These sampling jars were carefully to the laboratory. Standard morphological keys described by Fonseka [24] were used for the identification of different dragonfly nymphs up to the species level. The identified nymphs were introduced into glass tanks (60 × 30 × 35cm) filled with pond and well water from the area at 1:1 ratio, ensuring that different species of dragonfly nymphs are maintained in separate holding tanks. Both protozoan and planktonic algae which are natural sources of food for the dragonfly nymphal stages were provided into the holding tanks via addition of pond water [25]. At least a single individual of each identified species was reared up to the adulthood within the glass tanks under uniform laboratory conditions, while providing stone surfaces near emergence period to further confirm the species identification. Bedjanič* et al.* [14] used it for species identification of emerging adult specimens.

## 3. Predation Experiments

### 3.1. Evaluation of the Predatory Efficacy of Different Dragonfly Species

Dragonfly nymphs of different species were reared up to the final instar stage and their body lengths were measured to the closest millimeter by using a ruler. A well grown individual of each species was introduced into separate holding tanks (maintained as described above) and was starved for 12 hours [13]. Hundred 4th instar larvae of* Aedes aegypti *reared under laboratory settings were introduced into each glass tank initially, while a similar glass tank without any dragonfly nymphs was used as the control. In case where more than 75% of* Aedes* larvae were predated, new batches of 4th instar larvae were introduced into the glass tanks to maintain the 100 individuals per tank larval density throughout a day. After completion of 24 hours, another 100* Ae. aegypti* larvae were introduced into each tank. The number of surviving mosquito larvae in each tank was enumerated at 1 hour intervals until 48 hours with minor modifications to the methodology described by Singh* et al.* [25] and Shad and Andrew [1]. The whole experiment was repeated for five times to maintain the accuracy of the findings.

### 3.2. Effect of the Nymphal Stage of the Dragonfly Nymph on the Predatory Efficacy

The dragonfly species with the highest nymphal predation rate were recognized from the above experiment (i.e.,* Anax indicus*). The total length of the dragonfly nymph, measured to the closest millimeter by using a ruler, was used to classify the nymphal stage into three classes as (a) initial stage (10-20 mm of total body length in the case of* Ana. indicus*); (b) medium stage (25-35 mm), and (c) matured stage (35–45 mm). The above setup was replicated five times with each of the three different body sized dragonfly nymphs of the species found to have the highest predation efficacy.

### 3.3. Data Interpretation and Statistical Analysis

The predation rates were calculated as the deducted product of remaining mosquito larvae from the initial/earlier surviving larvae. The predation rates of each studied dragonfly nymph on* Ae. aegypti* larvae were entered into a Microsoft Excel Work Sheet. The significance in the total and hourly average predation rates of dragonfly nymphs were statistically evaluated by using the General Linear Model (GLM) followed by Tukey's pairwise comparison in SPSS (version 23). The Bray Curtis Similarity based Cluster analysis followed by Analysis of Similarities (ANOSIM) (i.e., a nonparametric analog of MANOVA) was utilized to identify the overall clustering status of the studied dragonflies in terms of their predation patterns using the Plymouth Routines in Multivariate Ecological Research version 6 (PRIMER 6) software [26].

## 4. Results

### 4.1. The Predatory Efficacy of Nymphs of Different Dragonfly Species on Aedes aegypti Larvae

**Figure 2:**
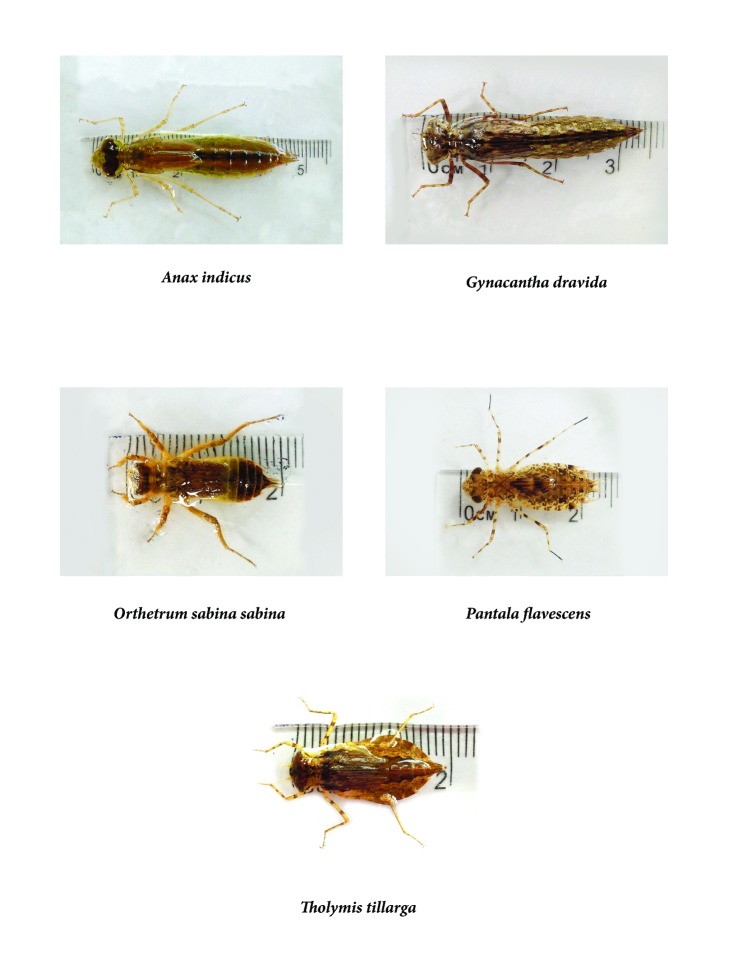
Different dragonfly larval species evaluated during the predation experiments.

Nymphs of five dragonfly species, namely,* Anax indicus, Gynacantha dravida, Orthetrum sabina sabina, Pantala flavescens, *and* Tholymis tillarga, *were identified from the field collections to be used for the predatory efficacy evaluation (Figure 2).* G. dravida* showed the longest length at the final instar stage (3.10 ± 0.18), while the lowest length of 1.55 + 0.12 was shown by* P. flavescens *(Table 1). Further, the results of the* Ae. aegypti* larval consumption by nymphs of different dragonfly species are summarized in Table 1. Among the five dragonfly species,* Ana. indicus* indicated the highest predation of 110 ± 7.14 (Mean ± SE)* Ae. aegypti* larvae within 24 hours, with an average hourly consumption rate of 4.58 ± 0.29 (Mean ± SE)* Ae. aegypti* larvae. On the other hand,* T. tillarga* denoted the lowest larval consumption of 23.47 ± 2.48 (Mean ± SE)* Ae. aegypti* larvae within 24 hours (Table 1). As suggested by the results of the General Linear Model, the predatory efficiencies of the five dragonfly species varied significantly (p<0.05 at 95% level of confidence).

Results of the Tukey's pairwise comparison (post hoc analysis) clearly denoted that the predatory efficacies of* P. flavescens *and* G. dravida* did not differ significantly (p>0.05 at 95% level of confidence), which differed significantly from that of* Ana. Indicus *(p<0.05). Meanwhile,* O. sabina sabina *and* T. tillarga *had the lowest predatory efficacies, which were statistically significant from the rest (p<0.05). Therefore, formation of three major clusters as* Anax indicus; P. flavescens *and* G. dravida *together; and* O. sabina sabina *and* T. tillarga *together could be recognized based on the predatory consumption of* Aedes aegypti* larvae as suggested by the GLM (Table 1).

**Figure 3:**
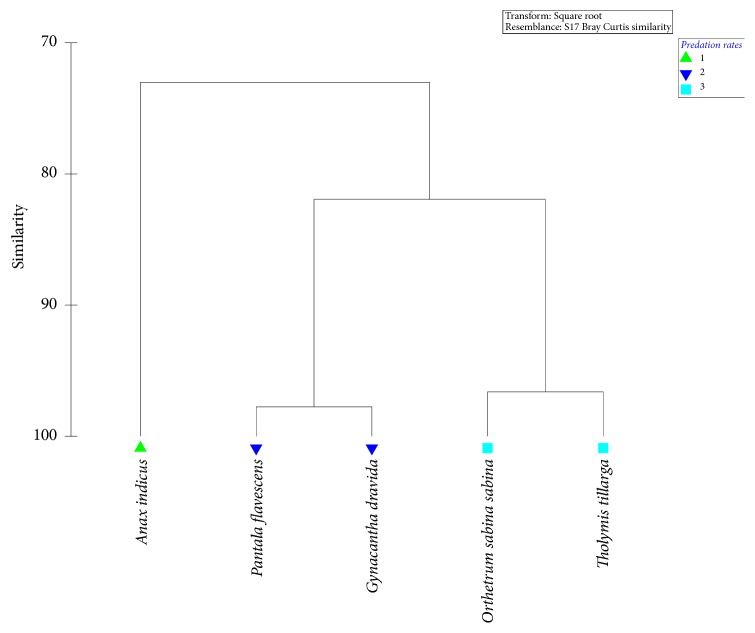
Dendrogram of the cluster analysis of the dragonflies in terms of the* Aedes* larval consumption patterns.

The dendrogram of the cluster analysis (based on Bray Curtis Similarity) also suggested the emergence of three clusters of dragonflies as* Ana. indicus* being the first cluster, while* P. flavescens* and* G. dravida* form the second (with a similarity of 96.6% among each other). Meanwhile,* O. sabina sabina* and* T. tillarga* created the third cluster sharing a similarity of 97.1% based on the total and hourly average predation rates of the studied dragonfly nymphs on* Ae. aegypti* larvae (Figure 3). Further, the dragonflies of second and third clusters shared a similarity of 81.4% among them in terms of their predatory rates. The clustering status of the above dendrogram was further confirmed to be statistically significant by the Global R value of 0.97 (p<0.05 at 95% level of confidence) from the Analysis of Similarities (ANOSIM). Therefore, the results of the General Linear Model regarding the significant variations of the predatory efficacies of the studied dragonflies were reassured by cluster analysis and ANOSIM.

### 4.2. The Predation Efficiency of Different Nymphal Stages of Anax indicus on Aedes aegypti Larvae

*Ana. indicus* nymphs reported the highest predatory efficacy on* Ae. aegypti* larvae from the current study. Therefore, the current study investigated the effect of larval body size of* Ana. indicus* (in terms of body length) on the predatory efficiency. The mean number of* Ae. aegypti* larvae consumed by* Ana. indicus* varied significantly according to the body size of the dragonfly larvae (p<0.05, at 95% level of significance).

The* Ana. indicus* nymphs with relatively larger body size (35-45 mm) indicated the highest predation, of 214.00 ± 13.20 as the total larval consumption within 24 hours along with, an hourly average of 8.92 ± 0.55* Ae. aegypti* larvae. Meanwhile, the smallest (25-35 mm)* Ana. indicus* larvae denoted the lowest predation rates of 52.87 ± 5.34* Ae. aegypti* larvae, under similar laboratory conditions (Table 2).

### 4.3. Temporal Variations in the Predation of Anax indicus on Ae. aegypti Larvae

#### 4.3.1. Diurnal Changes in Predation

**Figure 4:**
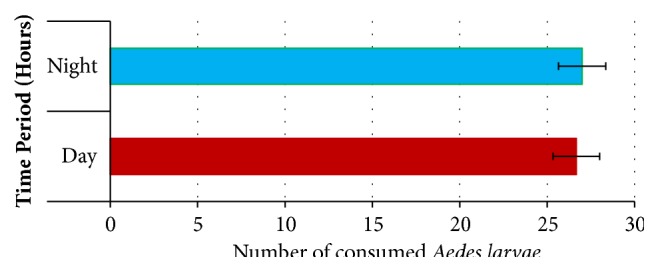
Diurnal changes in the* Aedes aegypti* mosquito larval consumption by* Anax indicus* (the best performer).

Due to being the best performer with the highest predation rates, the diurnal changes of the predatory action of* Ana. indicus* on* Ae. aegypti *mosquito were further investigated. As denoted by Figure 4, the predation rates of* Ana. indicus* were almost similar in both day (6.00 am to 6.00 pm) and night (6.00 pm to 6.00 am). However, it should be highlighted that only a limited number of replicates were carried out (n=5), which may be insufficient to arrive at a reasonable conclusion on the temporal variations of predatory rates of* Ana. indicus*, in terms of day and night.

### 4.4. Hourly Temporal Variation of the* Anax indicus* Larval Predation on* Aedes aegypti* Larva

**Figure 5:**
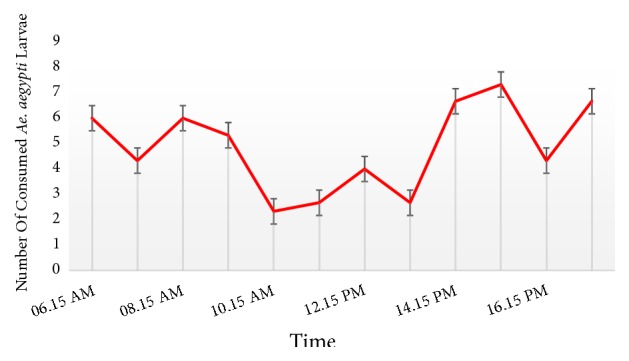
Temporal variation of the hourly average number of* Aedes aegypti* mosquito larvae consumed by* Anax indicus* (the best performer).

In case of the hourly temporal variations of the predation rates of* Ana. indicus* nymphs on the larvae of* Ae. aegypti*, two mild peaks of predation were observed in the dawn (6.00 a.m. to 9.00 a.m.) and dusk (3.00 p.m. to 6.00 p.m.) Among them, the highest predation rate was observed at the dusk. However, as indicated by Figure 5, significant variations in the hourly predation rates were not noted for* Ae. aegypti* larval consumption by* Ana. indicus.*

## 5. Discussion

Being one of the major health threats in Sri Lanka, dengue is challenging the health sector of the country with severe cyclic epidemics. The current vector control methods that mainly focus on the chemical based control of vectors with less emphasis on community involved vector management seem to have a limited success in managing the outbreaks of dengue [27]. Therefore, evaluation of the efficacy of novel vector control methods such as the use of different biological control agents is of essence to address the burning issue of dengue [1, 20, 25]. Dragonflies have shown promising results in this aspect and have been used in many neighboring counties with similar environmental and socioeconomic settings [12, 13, 25].

According to the current study, it was apparent that* Anax indicus*,* P. flavescens*,* G. dravida*,* O. sabina sabina,* and* T. tillarga* are active feeders and able to consume* Ae. aegypti* mosquito larvae in notable quantities under laboratory conditions. Results of the predation experiment indicated that* Ana. indicus* of Family Aeshnidae showed the highest predation.* Ana. indicus* generally oviposits in richly vegetated tanks and lakes in the dry lowlands of the island and in montane areas, where suitable still water habitats are present making it one of the abundant dragonfly species in rural and semiurban environments [28]. However, the availability of such habitats is limited in urban settings, highlighting the need for conservation of lowland wetlands and other stagnated aquatic habitats, to promote the natural distribution of this species as a biological control agent against dengue vectors. A study conducted by Becker* et al. *[29] has reported a similar predation potential of around 100 mosquito larvae by* Anisopteran* nymphs, suggesting it as an effective biological control agent. Thus, based on the current findings* Ana. indicus* could also be identified as an excellent candidate for biocontrol of dengue vectors.

Both* G. dravida *and* Ana. indicus* are members of Aeshnidae, which are commonly known as hawkers.* G. dravida* is distributed over low country, covering both wet and dry zones around swamps and tanks with weedy vegetation, and is often found inside houses as it is attracted to lights at night. The adults of those remain mostly active at dusk [28]. Therefore, this species has a high potential for predating upon both larvae and adults of* Aedes* mosquitoes, during its larval and adult stages in reducing* Aedes* populations.

*P. flavescens*, of the Family Libellulidae, which remains as the most common and widespread dragonfly species in Sri Lanka and possibly the most ubiquitous in the whole world [14], indicated the second highest predation rates of* Aedes* larvae. It is cosmopolitan and migrates in large numbers with the monsoon winds and are distributed all over Sri Lanka dwelling in open habitats including paddy fields, tanks, marshes, rivers, grasslands, lagoons, scrub forests, and large forest gaps [30]. Further, it is one of the most opportunistic breeders, which can breed in different conditions, ranging from muddy water pits to discarded containers in urban environments. In addition to the extreme dispersal ability,* P. flavescens* is characterized by a very rapid larval development rate, which often completes in just over one month [28]. Therefore,* P. flavescens, *which almost habitually breeds in every habitat, also remain as an ideal candidate to be used as a biological control agent against dengue vectors, due to the ability to adapt to various water bodies that are scattered within and around human settlements. In addition,* O. sabina sabina* and* T. tillarga *that showed relatively lower rates of predation in comparison to the other dragonfly nymphs also have a wider geographical distribution in Sri Lanka, occupying all types of wetlands found in the country [28].

Current study used 4th instar of* Ae. aegypti* mosquito larvae for all the predation experiments, since it remains as the larval stage with the highest body size. However, a previous study has reported that the predatory impact of* Bradinopyga geminata* on* Ae. aegypti* larvae was higher in 1st instar larvae than the other late instars, probably due to the smaller body size [12]. Another similar study has revealed that feeding rate of* B. contaminate* on aquatic stages of* Anopheles stephensi*,* Culex quinquefasciatus,* and* Ae. aegypti* also showed a similar trend, where the maximum predation was observed for the 1st instar larvae [25].

According to Córdoba-Aguilar and Lee [13], both smaller and larger dragonfly nymphs predate on bigger prey at high densities of prey. As shown by their findings,* Orthemis ferruginea* (fabricius) dragonfly larvae (Family: Libellulidae) predate on 4th instar* Ae. aegypti* mosquitoes than the 1st instar and pupae, especially by the smaller body sized predators. Although 4th instars are not larger than pupae, their shape might make them seem larger for predators. Therefore, a dragonfly's predation choice may be influenced by the shape and movement as well as the size of prey. The 4th instar mosquitoes are more active as well as larger than other three mosquito larval instar levels. Only smaller predators feed significantly more on the biggest prey at similar densities of different sizes of prey, while bigger dragonfly nymphs did not show a size preference [13]. This may be accounted by the high energy requirement of smaller sized dragonfly larvae due to their urge in completing the development stages in the life cycle.

However, in the current study,* Ana. indicus* with relatively larger body size (35-45 mm) indicated the highest predation of 214.00+13.20 as the total larval consumption. Several studies that evaluated the predation of* Orthemis *spp. have shown that dragonfly nymphs with large body size predate upon mosquito larvae with no preference for the immature stages of mosquito [31, 32]. Further, findings of Chatterjee* et al*. agree with the current findings by highlighting that small sized nymphs of* Brachytron pratense* were not interested to prey upon fourth instar stage of mosquito larvae in laboratory, without prolonged starvation [33]. Therefore, the variations in the gape size of the dragonfly nymphs that occur with advancement of age may have resulted in the high predation of 4th instar larvae by larger body sized nymphs.

The diurnal variation in the predation of* Ana. indicus* dragonfly larvae on* Ae. aegypti* larvae did not differ significantly within the present study. The findings of the current study stand in line with that of Córdoba-Aguilar and Lee, who also showed that there is no significant difference in the predation in dark and light conditions of* Orthemis ferruginea* against* Ae. aegypti* larvae [13]. Interestingly, in the present study, the predation of* Ana. indicus* was slightly higher at dawn and dusk, while predation was lower at the mid-day period. Further, the highest predation rate was observed at the dusk, regardless of the absence of significant variations in the hourly predation rates. Similar findings have been reported by Venkatesh and Tyagi, whereby the percentage consumption of* Ae. aegypti* larvae by* Bradinopyga geminate* remained with no marked diurnal variation [12]. Moreover, a relatively high predation rate of mosquito larvae in darkness (which was not statistically significant) has been evidenced for* Tramea* spp. [31]. On the other hand, a previous study conducted by Mandal* et al.* has highlighted a significant diurnal variation of* Cx. quinquefasciatus* predation, indicating a lower predation rate at night [19].

Usually, dragonfly nymphs belonging to the genus* Anax* are highly adapted surface feeders, and their diurnal rhythm of feeding has been attributed to various facts in the literature [24]. The flicker frequency for response of those is found to be associated with the intensity of light, while the mean critical illumination is progressively shifted towards higher intensities at the lower ambient temperatures [28]. Therefore, the progressive increase in significance of the eyes might be expected to have affected the diurnal rhythm of feeding activity, regardless of the statistically nonsignificance of predation rates. Further, the genus* Anax* is known to indicate a circadian rhythm of locomotory activity, which is entrained by the interchanging light-dark cycle. They have been reported to walk slowly over the bottom at night, instead of moving by jet propulsion during day time, enabling them to have a more probability of finding mosquito larvae as prey [24].

A number of parameters have been found to influence the predation rates and biocontrol efficacy of dragonfly nymphs on* Aedes* larvae. Increasing larval size and instar stage of the prey (*Aedes* larvae) [12], size, maturity stage, and energy requirements of the predator (dragonflies) along with and other physiological attributes of predators [34–36] tend to affect the above said predation rates. Further, several environmental factors such as temperature, illumination, container size, and foraging area could also affect the mosquito larval consumption by dragonflies [1]. The survival, development, and recruitment levels of mosquitoes (from immature stages to adult vector populations) are highly influenced by predaceous insects, especially by dragonflies. Therefore, detaining of the immature stages of vectors in most of the aquatic habitats has maintained vector densities below the critical thresholds, leading to control of epidemic incidence [37–39]. Several semifield studies have highlighted the efficacy of using different dragonfly larval species as a biocontrolling agent to suppress variety of vector mosquito populations such as* Anopheles subpictus *[37],* Ano. stephensi *[39],* Ae. aegypti *[12, 20, 39], and* Cx. quinquefasciatus *[19]. Based on the findings of the present study,* Anax indicus *that denoted the highest predation rate for* Ae. aegypti *is recommended as a biocontrol agent for controlling of dengue through introduction into the field. Further,* P. flavescens* which was characterized by the second highest biocontrol efficacy could also be focused on which may be as a better candidate for field trials due to its wide geographical distribution and ability of completing the life cycle within a relatively short period in different habitats, especially in urban environments [24, 28].

Use of dragonflies as a biological control agent against* Aedes* vectors would essentially be a key solution to control dengue, due to their ability to kill target species, safety awarded to nontarget organisms, easy application in the field, inexpensive production, lack of infectivity, and no pathogenicity in mammals including man [12, 13, 40]. The success and sustainability of such biocontrol activities heavily depend upon the land use practices and development activities. Poorly planned development activities that occur within urban and semiurban areas in the country may directly contribute to the occurrence of breeding sites for dengue vectors, while diminishing the quality of potential breeding habitats of dragonflies [27, 40]. Therefore, in order to reduce population of* Aedes* mosquitoes through biocontrolling by dragonfly larvae, it is essential to conserve breeding habitats such as paddy fields, tanks, marshes, rivers, grasslands, lagoons, scrub forests, and large tracts of forest [29]. However, facilitation of the* P. flavescens *populations might be an ideal solution, due to its ability of breeding in numerous urban habitats scattered within and around human settlements. Therefore, it is suggested to conduct further research on the biocontrol efficacy of* Ana. indicus *and* P. flavescens *under semifield and field conditions as a pioneering project to evaluate the practical feasibility of suppressing* Aedes* vector populations by the use of dragonflies within Sri Lanka.

## 6. Conclusions

The predatory efficacy of the studied dragonflies on* Ae. aegypti* larvae differed significantly (p<0.05, at 95% level of significance).* Ana. indicus* showed the highest predation efficiency (110±7.14 per day) followed by* P. flavescens *(54.07±5.15). The predation efficiency of* Ana. indicus* significantly varied according to the maturity level (body size) of the dragonfly nymphs (p<0.05), whereby larger body sized dragonfly nymphs indicated the maximum predation rate of 214.00± 13.20* Ae. aegypti* larvae within 24 hours. Regardless of the absence of statistically significant variations in the diurnal variations in predation rates, a relatively high predation was observed at dusk.

In Sri Lanka,* P. flavescens *and* Ana. indicus* indicate relatively wider distribution along with high feasibility for adaptation. Therefore, the study recommends* Ana. indicus *and* P. flavescens *as potential candidates for a field trial of biological control of dengue via suppressing of vector populations. It can be recommended for the authorities working for dengue control in Sri Lanka to incorporate the introduction and facilitation of the dispersion of above dragonfly species as key steps in Integrated Vector Management (IVM) approaches to control the incidence of dengue viral outbreaks.

## Figures and Tables

**Table 1:** Mean number of *Aedesaegypti* larvae consumed by different dragonfly species in 24 hours.

**Dragonfly Species**	**Mean Length**	**Mean Number of * Aedes aegypti* larvae consumed by a dragonfly larva**	
		**Total number of larvae consumed within 24 hours**	**Average number of larvae consumed within 1 hour**
*Anax indicus*	2.87 ± 0.10 (2.77 – 2.97)	110.00 ± 7.14 ^a^ (102.86 - 117.14)	4.58 ± 0.29 ^a^ (4.29 - 4.87)
*Pantala flavescens*	1.55 ± 0.12 (1.43 – 1.67)	54.07 ± 5.15 ^b^ (48.92 - 59.22)	2.31 ± 0.22 ^b^ (2.09 - 2.53)
*Gynacantha dravida*	3.10 ± 0.18 (2.92 – 3.28)	49.00 ± 11.89 ^b^ (37.11 - 60.89)	2.20 ± 0.49 ^b^ (1.71 - 2.69)
*Orthetrum sabina sabina*	1.60 ± 0.18 (1.42 – 1.78)	26.87 ± 2.89 ^c^ (23.98 - 29.76)	1.09 ± 0.12 ^c^ (0.97 - 1.21)
*Tholymis tillarga*	1.57 ± 0.09 (1.48 – 1.66)	23.47 ± 2.48 ^c^ (20.99 - 25.95)	0.95 ± 0.10 ^d^ (0.85 - 1.05)

**Note:** values are Mean ± SE, range in parenthesis. Different superscript letters in a column show significant differences (p< 0.05) as suggested by General Linear Modelling followed by the Tukey's pair wise comparison at 95% level of significance.

**Table 2:** Mean number of *Aedes aegypti* larvae consumed by *Anax indicus* (the best performer, in terms of larval predation) with different body size categories.

**Body size/Maturity categories**	**Mean Number of * Aedes aegypti* larvae consumed by ** ***Anax indicus***	
	**Total number of larvae consumed within 24 hours**	**Average number of larvae consumed within 1 hour**
Large/Initial (35-45 mm)	214.00 ± 13.20 ^a^ (200.80 – 227.20)	8.92 ± 0.55 ^a^ (8.37 – 9.47)
Medium/Medium (25-35 mm)	110.00 ± 7.14 ^b^ (102.86 – 117.14)	4.58 ± 0.30 ^b^ (4.28 – 4.88)
Small/Matured (10-20 mm)	52.87 ± 5.34 ^c^ (47.53 – 58.21)	2.20 ± 0.22 ^c^ (1.98 – 2.42)

**Note:** values are Mean ± SE, range in parenthesis. Different superscript letters in a column show significant differences (p< 0.05) as suggested by General Linear Modelling followed by the Tukey's pair wise comparison at 95% level of significance.

## Data Availability

The data used to support the findings of this study are included within the article.

## Abbreviations

ANOSIM: Analysis of Similarities
DF: Dengue Fever
DHF: Dengue Haemorrhagic Fever
GLM: General Linear Model
IIT: Incompatible Insect Technique
IVM: Integrated Vector Management
IRS: Indoor Residual Spraying
MANOVA: Multivariate Analysis of Variance
MOH: Medical Officer of Health
PRIMER: Plymouth Routines in Multivariate Ecological Research
SIT: Sterile Insect Technique
SPSS: Statistical Package for the Social Sciences
WHO: World Health Organization.
