# Corrigendum to “Larvicidal Potential of Five Selected Dragonfly Nymphs in Sri Lanka over* Aedes aegypti* (Linnaeus) Larvae under Laboratory Settings”

**DOI:** 10.1155/2019/3950875

**Published:** 2019-02-05

**Authors:** Chathurika Samanmali, Lahiru Udayanga, Tharaka Ranathunge, Sandun J. Perera, Menaka Hapugoda, Chathura Weliwitiya

**Affiliations:** ^1^Department of Natural Resources, Faculty of Applied Sciences, Sabaragamuwa University of Sri Lanka, Sri Lanka; ^2^Molecular Medicine Unit, Faculty of Medicine, University of Kelaniya, Sri Lanka; ^3^Department of Bio-Systems Engineering, Faculty of Agriculture & Plantation Management, Wayamba University of Sri Lanka, Sri Lanka; ^4^HELPO Eco Green Ltd., Talbot Town, Galle, Sri Lanka

In the article titled “Larvicidal Potential of Five Selected Dragonfly Nymphs in Sri Lanka over* Aedes aegypti* (Linnaeus) Larvae under Laboratory Settings” [1], the second and third affiliations were incorrect. The corrected affiliations are shown above.
